# Following intravenous thrombolysis, the outcome of diabetes mellitus associated with acute ischemic stroke was predicted via machine learning

**DOI:** 10.3389/fphar.2025.1506771

**Published:** 2025-01-27

**Authors:** Xiaoqing Liu, Miaoran Wang, Rui Wen, Haoyue Zhu, Ying Xiao, Qian He, Yangdi Shi, Zhe Hong, Bing Xu

**Affiliations:** ^1^ Shenyang Tenth People’s Hospital, Shenyang Medical College, Shenyang, China; ^2^ The First Hospital of China Medical University, Shenyang, China; ^3^ Shenyang First People’s Hospital, Shenyang Medical College, Shenyang, China

**Keywords:** acute ischemic stroke (AIS), diabetes, thrombolytic, XGB, SHAP, 90-day MRS

## Abstract

This cohort study aimed to evaluate the prognostic outcomes of patients with acute ischemic stroke (AIS) and diabetes mellitus following intravenous thrombolysis, utilizing machine learning techniques. The analysis was conducted using data from Shenyang First People’s Hospital, involving 3,478 AIS patients with diabetes who received thrombolytic therapy from January 2018 to December 2023, ultimately focusing on 1,314 patients after screening. The primary outcome measured was the 90-day Modified Rankin Scale (MRS). An 80/20 train-test split was implemented for model development and validation, employing various machine learning classifiers, including artificial neural networks (ANN), random forest (RF), XGBoost (XGB), and LASSO regression. Results indicated that the average accuracy of the XGB model was 0.7355 (±0.0307), outperforming the other models. Key predictors for prognosis post-thrombolysis included the National Institutes of Health Stroke Scale (NIHSS) and blood platelet count. The findings underscore the effectiveness of machine learning algorithms, particularly XGB, in predicting functional outcomes in diabetic AIS patients, providing clinicians with a valuable tool for treatment planning and improving patient outcome predictions based on receiver operating characteristic (ROC) analysis and accuracy assessments.

## Introduction

Stroke is one of the leading causes of death and disability worldwide (Benjamin et al., 2018) and has become the primary cause of death in China, imposing a significant social and economic burden on society and families (Zhou et al., 2019). Acute ischemic stroke (AIS) accounts for approximately 69.6%–70.8% of all stroke cases (Wang D. et al., 2017; Wang W. et al., 2017). Alteplase (rt-PA), when administered within 4.5 h of symptom onset, has been shown to improve long-term outcomes in stroke patients. Furthermore, alteplase is the only pharmacological intervention approved for the treatment of acute ischemic stroke (Zeinhom et al., 2024).

In addition, diabetes mellitus is a prevalent chronic disease and a recognized risk factor for stroke (Visseren et al., 2021). The prevalence of diabetes among all types of stroke is 28%. Studies have shown that ischemic stroke (33%) is more common than hemorrhagic stroke (26%) (Lau et al., 2019). Among patients with ischemic stroke, those with diabetes tend to be relatively younger and have more comorbidities than nondiabetic patients do (Echouffo-Tcheugui et al., 2018). In addition, people with diabetes are at a greater risk of experiencing a recurrence of stroke than are those without diabetes (Zhang et al., 2021).

Diabetes mellitus is a significant risk factor affecting the prognosis of patients with AIS who undergo intravenous thrombolysis with recombinant tissue plasminogen activator (rt-PA) (Jian et al., 2023). Although previous studies have indicated that various risk factors influence the prognosis of patients with diabetes mellitus and AIS following intravenous thrombolytic therapy, there has been a lack of research utilizing machine learning methods to predict outcomes in patients with such comorbidities. Therefore, our objective was to develop and assess the applicability of machine learning (ML) models for predicting prognosis after intravenous thrombolysis in patients with diabetes mellitus and AIS. Additionally, we analyzed and highlighted the importance of the input variables to identify the most significant predictors of patient prognosis.

## Methods

### Standard protocol approval, registration, and patient consent

This study was designed as an observational, single-center, retrospective cohort study that included data from the follow-up center of Shenyang First People’s Hospital. The primary objective of this study was to assess and compare the efficacy of different machine learning models in predicting the prognosis of ischemic stroke in patients with diabetes following thrombolytic therapy. To ensure ethical considerations and maintain research integrity, this study was officially approved by the Research Ethics Committee of Shenyang First People’s Hospital (Grant No. 2023SYKYPZ58).

### Study design and population

Patients with acute ischemic stroke who were admitted to our hospital had received rt-PA thrombolytic therapy within 4.5 h were eligible for the study. All patients were evaluated and managed according to the criteria outlined in institutional stroke protocols, adhering to both international and domestic guidelines. In addition to laboratory tests, MRS is assessed in patients with acute ischemic stroke 3 months after the onset of symptoms.

The inclusion criteria for this study were as follows: 1) patients who experienced consecutive ischemic strokes between 2018 and 2023; 2) patients with a documented history of diabetes mellitus or those exhibiting fasting blood glucose ≥100 mg/dL or HbA1c ≥ 5.7%11; and 3) individuals who received rt-PA therapy within 4.5 h of symptom onset. Participants were excluded on the basis of the following criteria: 1) underwent urokinase thrombolytic therapy; 2) received stent therapy; 3) underwent balloon therapy; 4) lacked 90-day MRS; 5) other surgical procedures; and 6) individuals without a history of diabetes mellitus, fasting blood glucose measurements, or HbA1c laboratory markers. In addition, in order to deal with missing values in the data, we use the MICE (Multiple Imputation Chain Equation) method (Supplementary Table 4).

### Clinical variables

Data on baseline and demographic factors, including patient age and sex, were collected. The clinical factors included the National Institutes of Health Stroke Scale (NIHSS) score and stroke subtype, as classified by the Acute Stroke Treatment (AIS) classification in the ORG 10172 trial (TOAST). Additionally, data on vascular risk factors, including systolic blood pressure and smoking status, were gathered. Laboratory results, which included measurements of weight, fasting blood glucose (FBG), white blood cell count, blood platelet count, hemoglobin, HbA1c, cholesterol (CHOL), albumin (ALB), globulin (GLOB), albumin-to-globulin ratio (AG), alanine aminotransferase (ALT), aspartate aminotransferase (AST), creatinine, stress hyperglycemia (SHR), DL-homocysteine, and hypersensitive C-reactive protein, were also obtained. The outcomes were assessed at 3 months via the modified Rankin scale.

### Primary results

The clinical outcomes of the patients were monitored 3 months after the onset of symptoms. Functional outcomes were assessed via the 90-day MRS, a widely recognized instrument for measuring disability and evaluating recovery post-stroke. Two definitions of a favorable functional outcome were established: MRS of 0–1 and MRS of 0–2. The primary outcome was defined as 90-day MRS of 0–1, indicating that patients with 90-day MRS ranging from two to six were associated with a poor prognosis, whereas those with 90-day MRS of 0–1 were associated with a favorable prognosis. The secondary outcome was defined as a 90-day MRS of 0–2, with patients scoring between three and six reflecting a poor prognosis, whereas those with 90-day MRS of 0–2 demonstrated a good prognosis. All the data were collected by trained study coordinators who remained blinded to the baseline characteristics of the subjects.

### Machine learning model development

A total of 38 clinical and laboratory indicators were utilized in the development of an ML model aimed at predicting the prognosis of patients with comorbidities following intravenous thrombolysis. Clinical and laboratory indicators using p < 0.05 were used as inputs for machine learning. Experiments were subsequently conducted employing four machine learning classifiers, namely, LASSO regular logistic regression (LASSO) (Kang et al., 2021), random forest (RF) (Jin et al., 2023), extreme gradient boosting (XGB) (Lei et al., 2023), and artificial neural network (ANN) (Chun-An Cheng and Hung-Wen Chiu, 2017), to construct a proprietary model for predicting each study outcome. In this investigation, hierarchical k-fold cross-validation, class weighting, and random search techniques were implemented to mitigate overfitting and optimize the model. Initially, the dataset was partitioned into a training set and a test set in an 80:20 ratio, comprising 1,051 and 263 participants, respectively. The training dataset was further divided into five segments, and cross-validation was performed by ensuring that each segment maintained the same class proportions. This cross-validation process helps prevent overfitting to specific datasets and fosters the development of a more generalized model. To address the issue of data imbalance, class weighting techniques were applied alongside focus loss and resampling methods. The categorical ratio for each outcome variable was determined on the basis of the 90-day MRS, which was calculated as the average loss of contribution.

In recent years, the enhancement of machine learning (ML) model performance has increased the significance of explainable artificial intelligence (XAI), which aims to elucidate model outcomes. Among the various methodologies employed, SHAP (SHapley Additive exPlanations) (Dewi et al., 2023) is utilized to quantitatively represent feature attribution. It is essential that feature attribution adheres to the principles of local accuracy, omission, and consistency. Notably, the SHAP value is the sole additive feature importance metric that fulfills these three criteria. Furthermore, the model’s impact is assessed by accounting for the interdependencies among variables. Consequently, the contribution of each variable to prognostic predictions can be visually analyzed. Therefore, the feature importance and relationships among prognostic correlation variables were derived through the application of SHAP values.

### Statistical analysis

Categorical variables are presented as counts with corresponding percentages (%), whereas continuous variables are reported as the means with standard deviations (SD) or medians with interquartile ranges (IQR). The Kolmogorov-Smirnov test was employed to verify the presence of a normal distribution. To evaluate differences between parametric continuous variables, the t-test was utilized; for nonparametric variables, the Mann‒Whitney U test was applied; the chi‒square test was used for categorical variables; and Fisher’s exact test was conducted for 2 × 2 contingency tables. No adjustments were made for multiple comparisons. A bilateral p value of less than 0.05 was considered statistically significant. The area under the ROC curve (AUC), accuracy, and F1 score for each machine learning model were computed to assess the performance of the developed machine learning model. All analyses were conducted via R version 4.4.0 and Python version 3.11.7. Optuna:1.4.0,TensorFlow version 2.6.0 and scikit-learn version 1.0.2 are used for model training. SHAP 0.40.0 is used to calculate the SHAP value.

## Results

### Baseline characteristics

**FIGURE 1:**
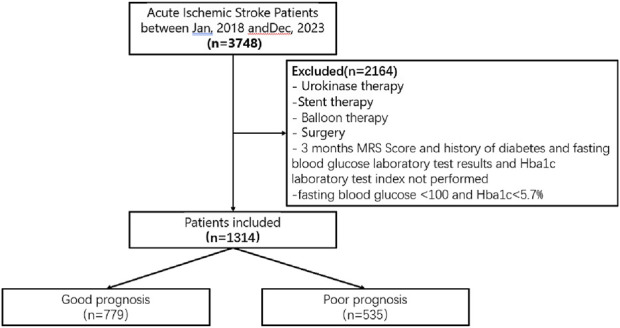
Study enrollment process.

During the study period, a total of 3,478 patients were admitted to the hospital for acute stroke, of which 1,314 patients were enrolled in this study (Figure 1). The mean age of the participants was 64.00 years (with a range of 58.25–71.00 years), and the duration from stroke onset to the return visit was 3 months. Within our cohort, the median NIHSS score was 3 (IQR 1–4). According to the MRS score, 779 of the 1,314 enrolled patients exhibited a favorable prognosis 3 months post-stroke. The baseline characteristics of the groups with good and poor prognoses are presented in Table 1. Poor prognosis was significantly associated with older age, a history of cardiac disease, hyperlipidemia, lower body weight, and a higher initial NIHSS score. Additionally, the group with poor prognostic outcomes had lower lymphocyte counts and albumin‒globulin ratios (AGs), as well as elevated neutrophil counts, blood platelet counts, total bilirubin (TBL), direct bilirubin (DBL), globulin (GLOB), creatinine, hypersensitive C-reactive protein, and DL‒homocysteine levels (Table 1). Therefore, we used these abnormal indicators as inputs to the machine learning model to verify which machine learning predicted the prognosis of patients with the highest accuracy (Supplementary Table 1).

**TABLE 1:** Demographic and clinical features and laboratory indices of diabetic patients receiving rt-PA thrombolytic therapy after stroke for primary outcome.

	Overall (1314)	Good prognosis (779)	Poor prognosis (535)	p	Test
Demographic characteristics					
Male (%) (man)	921 (70.1)	554 (71.1)	367 (68.6)		Exact
Age (median [IQR])	64.00 [58.25, 71.00]	63.00 [58.00, 69.00]	66.00 [60.00, 74.00]	**<0.001**	Nonnorm
Weigh (median [IQR])	70.00 [63.00, 78.00]	70.00 [65.00, 80.00]	70.00 [60.00, 75.50]	**0.003**	Nonnorm
Vascular risk factors					
Systolic pressure (mean (SD)	149.72 (18.22)	148.91 (18.15)	150.90 (18.26)	0.052	
Diastolic blood pressure (mean (SD)	85.99 (11.59)	85.97 (11.27)	86.02 (12.05)	0.942	
smoking (%)	752 (57.2)	429 (55.1)	323 (60.4)	0.061	Exact
History of heart failure (%)	1282 (97.6)	769 (98.7)	513 (95.9)	**0.002**	Exact
History of alcohol consumption (%)	929 (70.7)	545 (70.0)	384 (71.8)	0.498	Exact
diabetes (%)	634 (48.2)	376 (48.3)	258 (48.2)	1	Exact
Hyperlipidemia (%)	1125 (85.6)	653 (83.8)	472 (88.2)	**0.031**	Exact
Stroke characteristics					
Toast (%)				**<0.001**	Exact
LAA	977 (74.4)	568 (72.9)	409 (76.4)		
SAO	218 (16.6)	160 (20.5)	58 (10.8)		
CE	91 (6.9)	32 (4.1)	59 (11.0)		
SUE	27 (2.1)	18 (2.3)	9 (1.7)		
SOE	1 (0.1)	1 (0.1)	0 (0.0)		
NIHSS (median [IQR])	3.00 [2.00, 6.00]	3.00 [2.00, 4.00]	5.00 [3.00, 10.00]	**<0.001**	Nonnorm
Laboratory findings					
FBG (mean (SD))	9.01 (4.00)	8.86 (4.03)	9.24 (3.94)	0.088	
White blood cell count (mean (SD))	8.38 (18.53)	7.64 (2.20)	9.45 (28.90)	0.081	
Lymphocyte count (mean (SD))	1.80 (0.63)	1.86 (0.61)	1.70 (0.64)	**<0.001**	
Neutrophil count (mean (SD))	5.44 (2.32)	5.14 (2.05)	5.88 (2.61)	**<0.001**	
RBC (mean (SD))	4.58 (1.29)	4.57 (0.64)	4.59 (1.86)	0.865	
Blood platelet count (mean (SD))	142.86 (108.62)	130.03 (107.17)	161.55 (108.09)	**<0.001**	
Hemoglobin (mean (SD))	155.80 (84.66)	158.26 (89.89)	152.22 (76.35)	0.204	
HbA1c (mean (SD))	7.36 (1.74)	7.33 (1.69)	7.41 (1.81)	0.39	
CHOL (mean (SD))	4.96 (1.21)	4.99 (1.20)	4.92 (1.22)	0.283	
HDL-C (mean (SD))	1.02 (0.26)	1.02 (0.27)	1.03 (0.25)	0.893	
LDL-C (mean (SD))	3.23 (0.83)	3.23 (0.80)	3.24 (0.87)	0.756	
TG (mean (SD))	1.93 (1.48)	1.99 (1.49)	1.84 (1.46)	0.066	
TBIL (mean (SD))	16.73 (6.88)	16.22 (6.23)	17.48 (7.67)	**0.001**	
DBIL (mean (SD))	2.95 (1.62)	2.87 (1.55)	3.07 (1.71)	**0.022**	
TP (mean (SD))	66.78 (5.78)	66.60 (5.55)	67.03 (6.10)	0.186	
ALB (mean (SD))	40.90 (3.56)	41.03 (3.46)	40.71 (3.70)	0.111	
GLOB (mean (SD))	25.88 (4.69)	25.57 (4.66)	26.32 (4.70)	**0.004**	
AG (mean (SD))	1.65 (0.41)	1.68 (0.46)	1.60 (0.32)	**0.001**	
ALT (mean (SD))	22.55 (17.67)	22.69 (16.26)	22.35 (19.55)	0.729	
AST (mean (SD))	21.84 (13.90)	21.98 (14.64)	21.63 (12.75)	0.656	
Creatinine (mean (SD))	73.38 (32.19)	70.86 (26.78)	77.06 (38.46)	**0.001**	
SHR (mean (SD))	1.20 (0.36)	1.19 (0.36)	1.23 (0.36)	**0.025**	
Blood urea nitrogen (mean (SD))	5.88 (1.88)	5.82 (1.87)	5.96 (1.91)	0.191	
Hypersensitive C-reactive protein (mean (SD))	6.69 (13.76)	6.04 (13.29)	7.64 (14.37)	**0.038**	
DL-Homocysteine (mean (SD))	17.82 (10.94)	17.22 (10.88)	18.68 (10.97)	**0.017**	

Abbreviations: TOAST, Trial of ORG, 10172 in Acute Stroke Treatment, LAA, large artery atherosclerosis; SVO, Small vessel occlusion; CE, Cardioembolism; OD, Other determined; UD, undetermined, NIHSS NIH, Stroke Scale score; FBG, fasting blood glucose; CHOL, Cholesterol; HDL-C, High-density lipoprotein cholesterol; LDL-C, Low-density lipoprotein cholesterol; TG, triglyceride; TBIL, total bilirubin; DBIL, direct bilirubin; TP, total protein; ALB, albumin; GLOB, globulin; AG, Albumin-to-globulin ratio,ALT, alanine aminotransferase; AST, aspartate transaminase; SHR, stress hyperglycemia.

The bold values indicates that the P-value is less than 0.05.

### Predictive models

**FIGURE 2:**
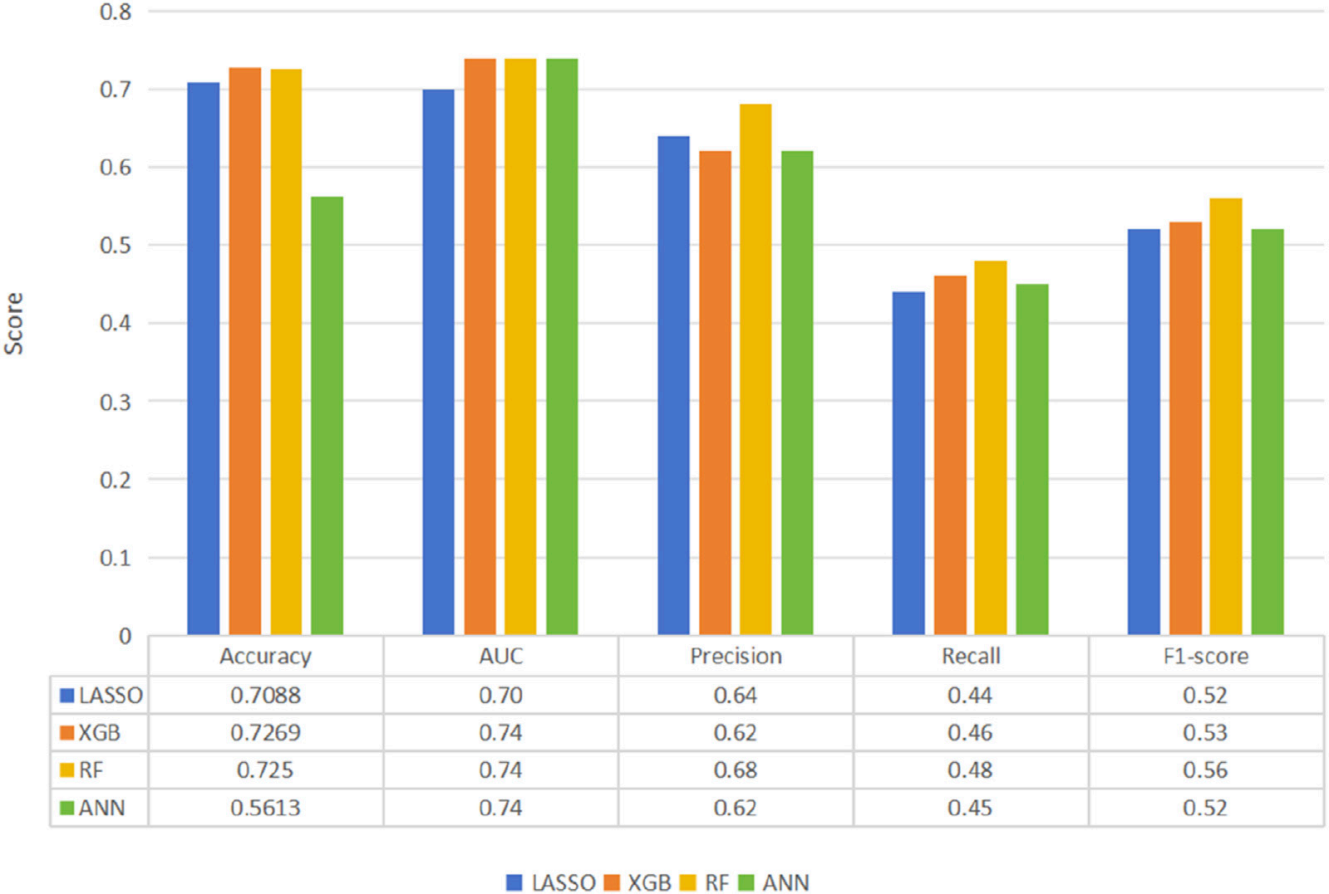
Comparison of machine learning model performance for the prediction of thrombolytic therapy prognosis according to the primary outcome of the best-performing model, XGB.

**FIGURE 3:**
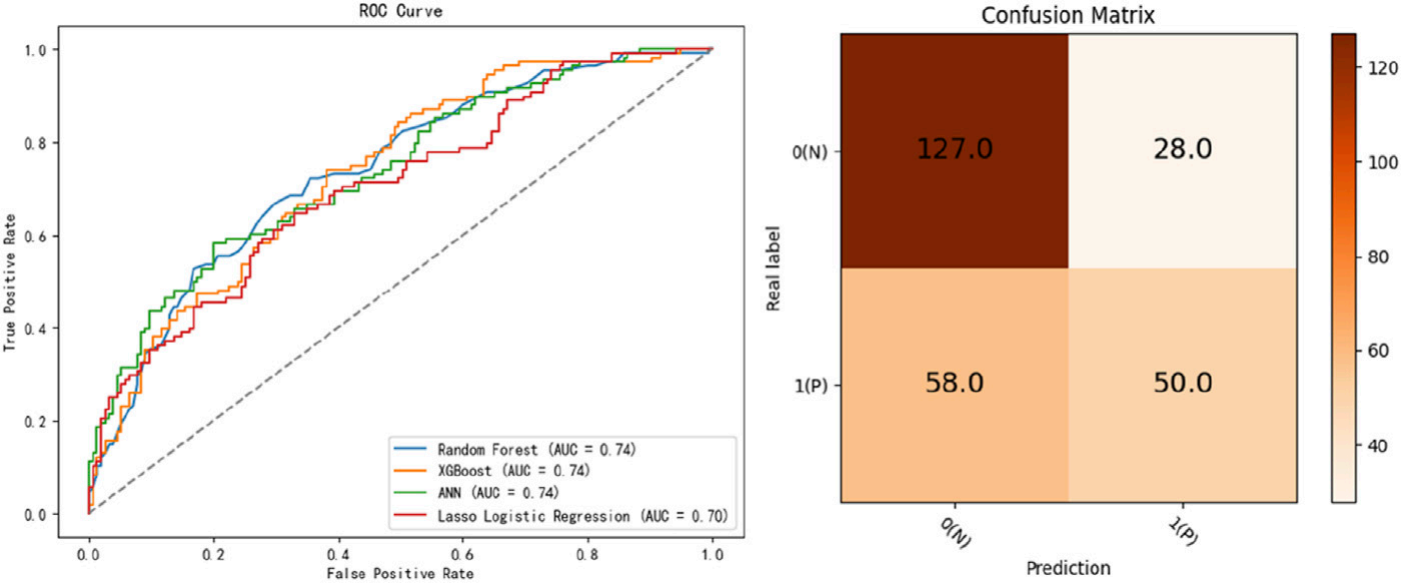
Receiver operating characteristic curves for the developed machine learning models and the confusion matrix of the best-performing model, XGB.

Four predictive models, namely, the LASSO, RF, XGB, and ANN models, were developed to assess patient prognosis. The mean predicted prognostic accuracies were 0.7269 (±0.0317) (XGB), 0.7088 (±0.03653) (LASSO), 0.7250 (±0.0201) (RF)and 0.5613 (±0.0252) (ANN) (Figure 2). Additionally, the receiver operating characteristic (ROC) curves for the best-performing models, along with their corresponding confusion matrices, are presented in Figure 2. Notably, the XGB model demonstrated the highest average accuracy, followed by RF, LASSO, and ANN (Figure 3).

### Functional importance

**FIGURE 4:**
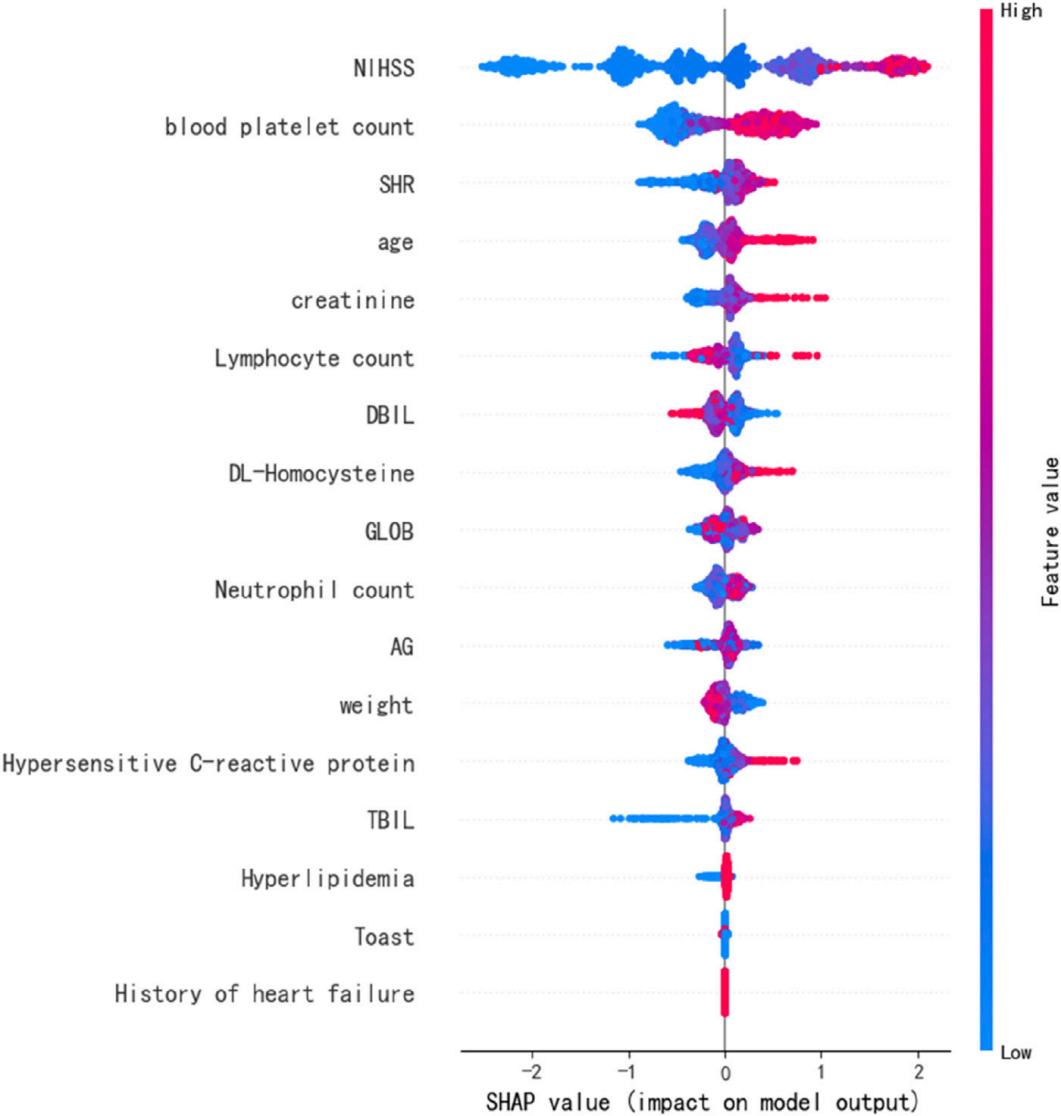
SHapley additive exPlanation values of the best prediction model, XGB.

We identified significant variables for predicting prognosis through the SHAP values derived from the optimal predictive model, XGB. The most critical variables for assessing stroke prognosis include the discharge NIHSS score and blood platelet count. SHR ranks as the second most important prognostic predictor, followed by age,creatinine and lymphocyte count (Figure 4). Other notable features identified from the 3 ML models include weight, GLOB, neutrophil count, and TBIL (Supplementary Figure 1).

### Secondary outcomes

The 90-day MRS (0–2) was used as a secondary outcome measure. The factors that had a greater impact on the secondary outcome (p < 0.05) were selected as input items for machine learning (Supplementary Tables 2, 3). The average accuracy of the prognostic predictions via the 4 ML models was as follows: 0.8088 (0.0242) for XGBoost, 0.7888 (0.0203) for ANN, 0.7840 (0.0203) for RF, and 0.8030 (0.0184) for LASSO (Supplementary Figure 2). XGB exhibited the highest average accuracy, followed by LASSO, ANN and RF. The ROC curves for the best-performing models are presented in the supplementary figure. The area under the curve (AUC) for prognostic prediction was 0.76 for XGBoost, 0.76 for RF, 0.74 for ANN and 0.72 for LASSO (Supplementary Figure 3).

## Discussion

This study aimed to develop and validate a machine learning (ML) model intended to predict the prognosis of diabetic patients following thrombolysis in cases of acute ischemic stroke. The results indicate that ML techniques, particularly the XGB model, can effectively predict patient outcomes post-thrombolysis. This advancement offers a novel tool for the management of individuals with diabetes mellitus and stroke. Furthermore, our findings provide empirical evidence supporting the application of ML technology in medical prognostication and suggest potential avenues for future research.

Among the 4 ML models examined in this study, the XGB model demonstrated the highest accuracy and the largest area under the curve (AUC), suggesting superior performance in predicting prognosis following thrombolysis in patients with diabetes mellitus and AIS. Since there is still some controversy about how to classify MRS to represent the prognosis of patients (Bala et al., 2023), we will determine the impact of two different grading methods on the prediction of machine learning models, and find that the XGB model performs well and accurately under different MRS grades. This finding aligns with the literature, as previous studies have indicated that XGB models are effective in various medical prediction tasks. For example, the prognosis of AIS patients was accurately forecasted via the XGB model, which exhibited high predictive performance on the basis of three key biomarkers: age, weight, and the NIHSS score (Chung et al., 2023). Consequently, our predictive analysis utilizing the XGB model identified several significant predictors, including stroke severity (NIHSS), blood platelet count,SHR,age, lymphocyte count, and creatinine. These predictors have been previously investigated in traditional models, with the NIHSS score recognized as a critical predictor of patient prognosis (Tanaka et al., 2013) in patients with acute ischemic stroke complicated by diabetes. Furthermore, research examining the relationship between blood platelet metrics (Chai et al., 2013) and prognosis has highlighted the significant influence of blood platelet reactivity (Harima et al., 2019) and aggregation on patient outcomes, underscoring the importance of incorporating the blood platelet count in functional outcome predictions. These findings substantiate the accuracy of the predictors identified in our XGB model and affirm their clinical relevance.

This study presents an innovative approach that integrates machine learning (ML) techniques with demographic information, past medical history, and laboratory data to predict patient prognosis following thrombolysis. This methodology contrasts with previous research that has relied predominantly on traditional statistical models or isolated variables. Additionally, we use the SHAP method to elucidate the contribution of each input variable to the predictive outcomes, thereby enhancing the explanatory power and transparency of our model. The SHAP values offer an intuitive framework for understanding the predictive logic of ML models, which is particularly crucial in the context of healthcare predictive modeling. For example, a study conducted by Lee et al. demonstrated that the XGB model could effectively predict factors influencing patient prognosis via SHAP values (Lee et al., 2023).

Nonetheless, several limitations are present in our study. First, the research is based on data from a single center, which may restrict the generalizability of the findings due to the specific patient population and standard of care at that institution. Similar to the limitations identified in our study, previous research has indicated that results derived from single-center data may lack broad applicability. Consequently, future investigations should aim for external validation across multiple centers and diverse patient cohorts to increase the generalizability and reliability of the model. Second, owing to high attrition rates and suboptimal follow-up after 1 year, we utilized the 90-day MRS as the primary outcome variable instead of the score obtained after 1 year. The correlation between carotid atherosclerosis and the 90-day MRS (Wu et al., 2024), as analyzed in the study by Wu et al., suggests that the 3-month MRS can serve as a predictor of patient prognosis; however, scores obtained after 1 year typically exhibit greater sensitivity and specificity. Therefore, future studies should endeavor to minimize follow-up attrition and utilize long-term outcome measures whenever feasible to comprehensively evaluate the long-term prognosis of patients. Additionally, while we have identified certain risk factors that can be managed through medication (e.g., blood platelet count and creatinine), there is limited evidence regarding the effectiveness of controlling these factors to enhance patient outcomes. Relying solely on the management of these risk factors may not be adequate to significantly improve patient outcomes; thus, a combination of comprehensive treatment strategies and individualized management may be necessary. Future research should explore the integration of machine learning prediction outcomes with comprehensive treatment strategies to achieve improved prognostic results.

In conclusion, our research demonstrates that machine learning (ML) techniques, particularly the XGBoost (XGB) model, are effective in predicting the prognosis following thrombolysis in patients with acute ischemic stroke who also have diabetes mellitus. This finding introduces a novel tool for the treatment and management of individuals with both diabetes and stroke. Future investigations should aim to validate these results in multicenter cohorts and further examine how comprehensive measures can be employed to enhance patient outcomes. By deepening our understanding and utilizing the predictive capabilities of ML models, we can anticipate improved outcomes in the treatment and management of stroke.

## Conclusion

To summarize, our study highlights the effectiveness of extreme gradient boosting (XGB) as a machine learning technique for predicting outcomes in patients with diabetes mellitus who experience acute ischemic stroke following intravenous thrombolysis. By utilizing the 90-day MRS (0–1) as the primary outcome, we compared the predictive accuracy of XGB with that of LASSO, RF, and ANN. XGB was ultimately selected for its superior performance, and we elucidated its advantages as a predictive tool. Additionally, we identified the risk factors that most significantly influenced the prognostic predictions by employing SHAP to interpret the XGB model’s predictions. This methodology has allowed us to clarify the key predictive determinants of thrombolytic therapy outcomes in patients with acute ischemic stroke and diabetes. Despite certain limitations, the XGB-based model presented in this study serves as a robust tool for clinicians, offering valuable insights for treatment planning and facilitating more accurate predictions of patient outcomes.

## Data Availability

The original contributions presented in the study are included in the article/Supplementary Material, further inquiries can be directed to the corresponding author.

## Abbreviations

AIS, Acute ischemic stroke; MRS, Modified Rankin scale; NIHSS, National Institutes of Health Stroke Scale; TOAST, Acute Stroke Treatment Classification in the ORG 10172 trial; LAA, Large artery atherosclerosis; SVO, small vessel occlusion; CE, cardioembolism; OD, Other determined; UD, Undetermined; FBG, fasting blood glucose; CHOL, Cholesterol; ALB, Albumin; GLOB, globulin; AG, Albumin-globulin ratio; ALT, Alanine aminotransferase; AST, Aspartate aminotransferase; SHR, Stress hyperglycemia; SHAP, SHapley Additive exPlanations; XGB, extreme gradient boosting; RF, random forest; ANN, artificial neural network; LASSO, LASSO regular logistic regression.
