# Supplementary material for: Following intravenous thrombolysis, the outcome of diabetes mellitus associated with acute ischemic stroke was predicted via machine learning
Source: Front Pharmacol. 2025 Jan 27;16:1506771. doi: 10.3389/fphar.2025.1506771 (PMC11808246; doi:10.3389/fphar.2025.1506771)
Supplement: Supplementary file 1 [file DataSheet1.docx]

**Supplemental Materials**

**Supplemental Figure 1.** The SHapley Additive exPlanations values of the machine learning models including ANN, LASSO, and RF for the prediction of primary outcomes following acute ischemic stroke in diabetic patients.


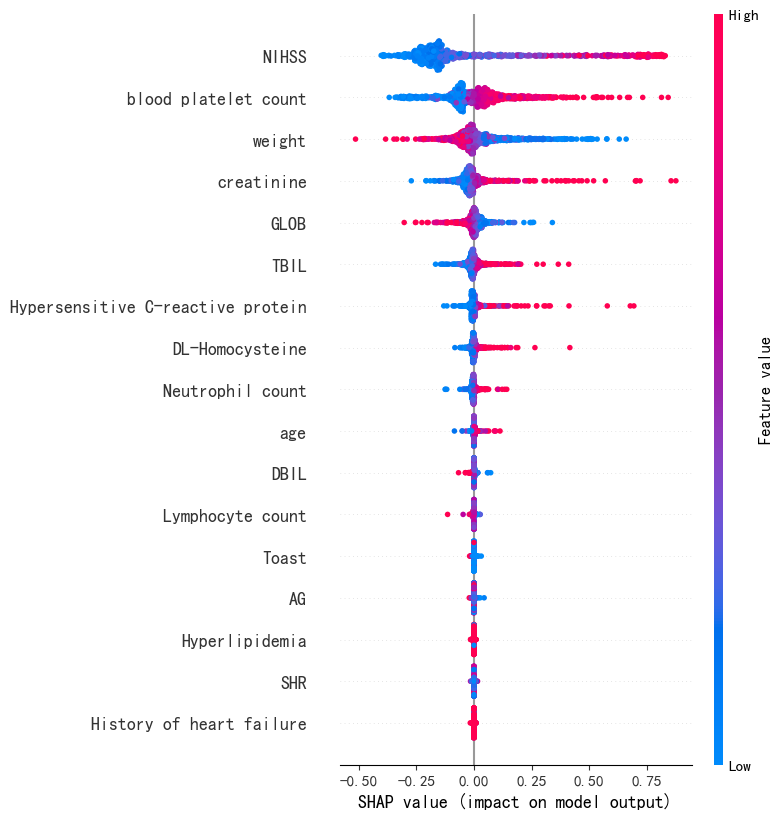


**LASSO**

**
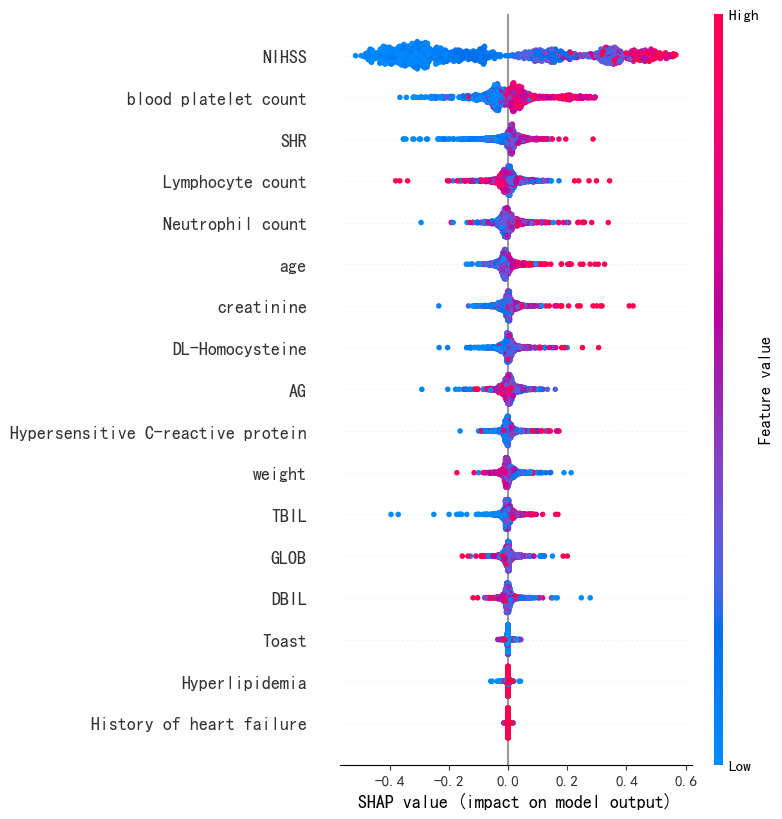
**

**RF**

**
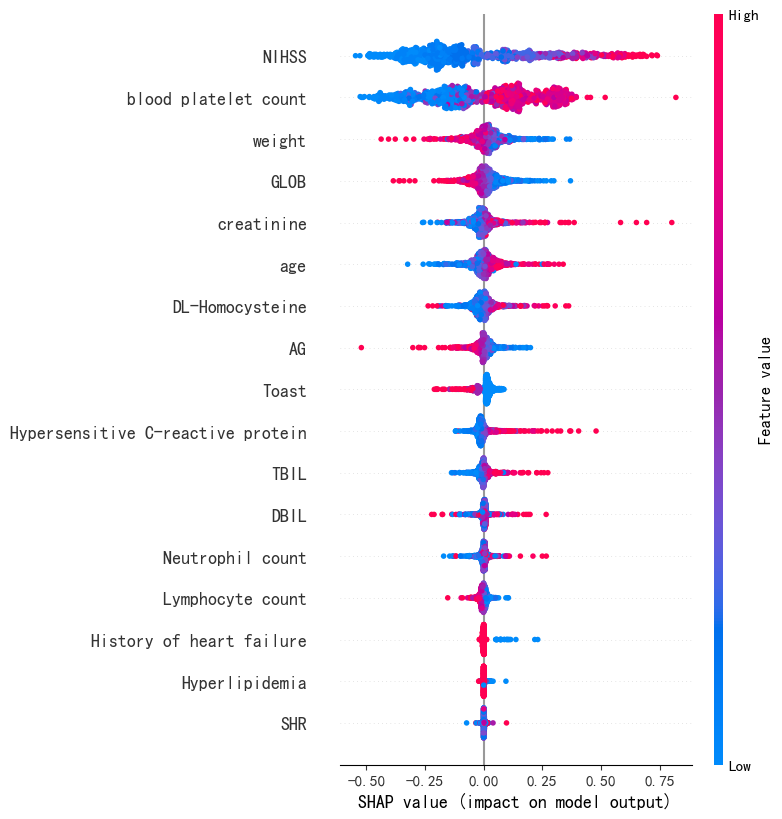
**

**ANN**

**Supplemental Figure 2 Comparison of machine learning model performance for the prediction of thrombolytic therapy prognosis according to the secondary outcome of the best‑performing model,RF**

**
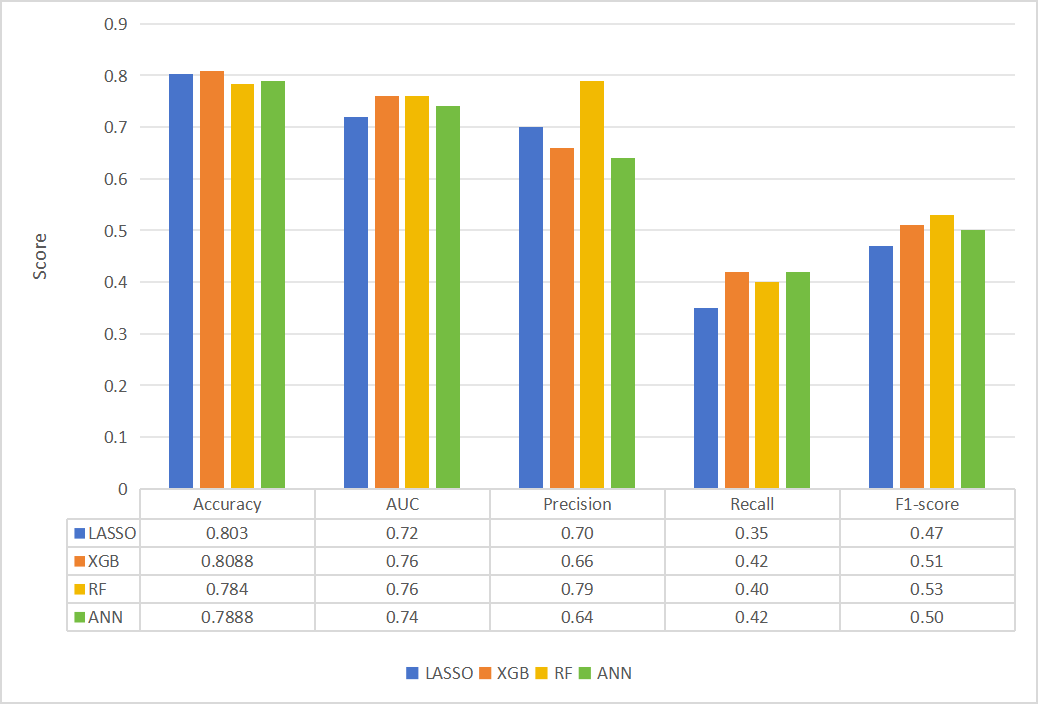
**

**Supplemental Figure 3.** Receiver Operating Characteristic curves for the developed machine learning models for the secondary outcomes.


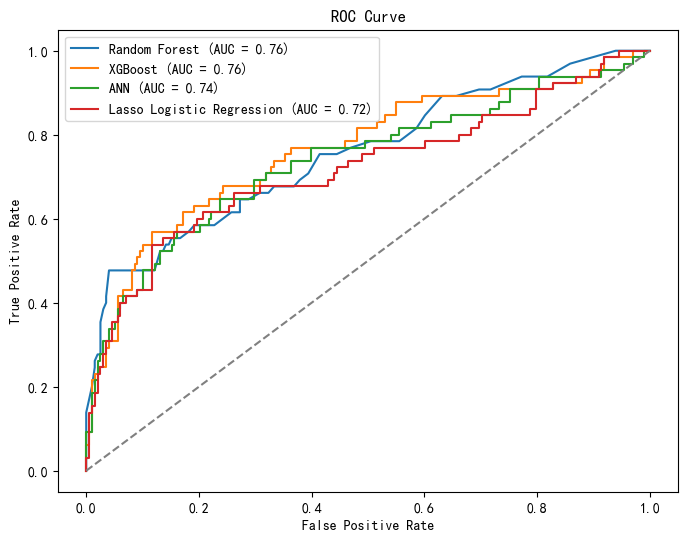


| **Supplemental Table 1. Input variables for machine learning model development for**  **primary outcome** | |
| --- | --- |
| **Demographic characteristics** | Age  Weight |
| **Vascular Risk factors** | History of heart failure |
|  | Hyperlipidemia |
| **Stroke Clinical Characteristics** | Discharge NIHSS |
|  | TOAST classification |
| **Laboratory findings** | Lymphocyte count |
|  | Neutrophil count |
|  | Blood platelet count |
|  | TBIL |
|  | DBIL |
|  | GLOB |
|  | AG |
|  | creatinine |
|  | SHR |
|  | Hypersensitive C-reactive protein |
|  | DL-Homocysteine |

*Abbreviations: TOAST Trial of ORG 10172 in Acute Stroke Treatment, TBIL Total bilirubin, DBIL Direct Bilirubin, GLOB Globulin, AG Albumin/Globulin, SHR Stress hyperglycemia.

**Supplemental Table 2: Demographic and clinical features and laboratory indices of diabetic patients receiving rt-PA thrombolytic therapy after stroke** **for secondary outcome.**

|  | **Overall**  **(1314)** | **Good prognosis**  **(779)** | **Poor prognosis**  **(535)** | **p** | **test** |
| --- | --- | --- | --- | --- | --- |
| **Demographic characteristics** |  |  |  |  |  |
| male (%)(man) | 921 (70.09) | 716 (71.96) | 205 (64.26) | **0.0113** | exact |
| age (median [IQR]) | 64.000 [58.250, 71.000] | 63.000 [58.000, 69.000] | 68.000 [62.000, 76.000] | **<0.0001** | nonnorm |
| weight(median [IQR]) | 70.000 [63.000, 78.000] | 70.000 [65.000, 79.000] | 70.000 [60.000, 75.000] | **0.0008** | nonnorm |
| **Vascular risk factors** |  |  |  |  |  |
| Systolic pressure (mean (SD) | 149.725 (18.217) | 148.780 (18.156) | 152.671 (18.120) | **0.0009** |  |
| Diastolic blood pressure (mean (SD) | 85.989 (11.586) | 85.952 (11.475) | 86.103 (11.942) | 0.8388 |  |
| smoking (%) | 752 (57.23) | 545 (54.77) | 207 (64.89) | **0.0014** | exact |
| History of heart failure (%) | 1282 (97.56) | 981 (98.59) | 301 (94.36) | **0.0001** | exact |
| History of alcohol consumption (%) | 929 (70.70) | 694 (69.75) | 235 (73.67) | 0.2032 | exact |
| diabetes (%) | 634 (48.25) | 481 (48.34) | 153 (47.96) | 0.9487 | exact |
| Hyperlipidemia (%) | 1125 (85.62) | 838 (84.22) | 287 (89.97) | **0.0141** | exact |
| **Stroke characteristics** |  |  |  |  |  |
| Toast (%) |  |  |  | **<0.0001** | exact |
| LAA | 977 (74.35) | 735 (73.87) | 242 (75.86) |  |  |
| SAO | 218 (16.59) | 199 (20.00) | 19 (5.96) |  |  |
| CE | 91 (6.93) | 39 (3.92) | 52 (16.30) |  |  |
| SUE | 27 (2.05) | 21 (2.11) | 6 (1.88) |  |  |
| SOE | 1 (0.08) | 1 (0.10) | 0 (0.00) |  |  |
| NIHSS (median [IQR]) | 3.000 [2.000, 6.000] | 3.000 [2.000, 4.000] | 7.000 [4.000, 13.000] | **<0.0001** | nonnorm |
| **Laboratory findings** |  |  |  |  |  |
| FBG (mean (SD)) | 9.013 (3.996) | 8.929 (4.079) | 9.273 (3.717) | 0.1812 |  |
| White blood cell count (mean (SD)) | 8.379 (18.530) | 7.634 (2.201) | 10.704 (37.355) | **0.01** |  |
| Lymphocyte count (mean (SD)) | 1.797 (0.629) | 1.841 (0.604) | 1.659 (0.685) | **<0.0001** |  |
| Neutrophil count (mean (SD)) | 5.441 (2.320) | 5.146 (2.039) | 6.360 (2.846) | **<0.0001** |  |
| RBC (mean (SD)) | 4.579 (1.285) | 4.568 (0.606) | 4.616 (2.381) | 0.5629 |  |
| Blood platelet count (mean (SD)) | 142.863 (108.616) | 138.983 (107.723) | 154.965 (110.655) | **0.0221** |  |
| Hemoglobin (mean (SD)) | 155.802 (84.660) | 156.988 (85.816) | 152.101 (80.968) | 0.3699 |  |
| HbA1c (mean (SD)) | 7.361 (1.740) | 7.346 (1.725) | 7.406 (1.790) | 0.5926 |  |
| CHOL (mean (SD)) | 4.959 (1.211) | 4.971 (1.199) | 4.921 (1.249) | 0.5283 |  |
| HDL-C (mean (SD)) | 1.024 (0.265) | 1.017 (0.263) | 1.047 (0.268) | 0.0789 |  |
| LDL-C (mean (SD)) | 3.232 (0.831) | 3.227 (0.803) | 3.248 (0.912) | 0.6978 |  |
| TG (mean (SD)) | 1.928 (1.477) | 2.019 (1.567) | 1.643 (1.108) | **0.0001** |  |
| TBIL (mean (SD)) | 16.733 (6.878) | 16.168 (6.173) | 18.496 (8.493) | **<0.0001** |  |
| DBIL (mean (SD)) | 2.951 (1.619) | 2.822 (1.490) | 3.353 (1.915) | **<0.0001** |  |
| TP (mean (SD)) | 66.778 (5.783) | 66.622 (5.577) | 67.264 (6.367) | 0.0842 |  |
| ALB (mean (SD)) | 40.903 (3.562) | 41.020 (3.450) | 40.536 (3.872) | **0.0348** |  |
| GLOB (mean (SD)) | 25.875 (4.687) | 25.602 (4.615) | 26.728 (4.813) | **0.0002** |  |
| AG (mean (SD)) | 1.645 (0.411) | 1.669 (0.434) | 1.571 (0.323) | **0.0002** |  |
| ALT (mean (SD)) | 22.552 (17.671) | 22.791 (15.930) | 21.805 (22.257) | 0.386 |  |
| AST (mean (SD)) | 21.841 (13.898) | 21.810 (13.590) | 21.936 (14.839) | 0.888 |  |
| Creatinine (mean (SD)) | 73.382 (32.187) | 72.430 (30.364) | 76.353 (37.201) | 0.0581 |  |
| SHR (mean (SD)) | 1.204 (0.357) | 1.192 (0.361) | 1.240 (0.340) | **0.0391** |  |
| Blood urea nitrogen (mean (SD)) | 5.878 (1.885) | 5.823 (1.848) | 6.051 (1.988) | 0.0603 |  |
| Hypersensitive C-reactive protein (mean (SD)) | 6.689 (13.756) | 6.103 (13.372) | 8.516 (14.763) | **0.0064** |  |
| DL-Homocysteine (mean (SD)) | 17.817 (10.935) | 17.676 (11.237) | 18.260 (9.938) | 0.4061 |  |

Abbreviations: TOAST Trial of ORG 10172 in Acute Stroke Treatment, LAA Large artery atherosclerosis, SVO Small vessel occlusion,CE Cardioembolism,OD Other determined,UD Undetermined, NIHSS NIH Stroke Scale score,FBG Fasting blood glucose, CHOL Cholesterol,HDL-C High-density lipoprotein cholesterol,LDL-C Low-density lipoprotein cholesterol,TG Triglyceride, TBIL Total bilirubin, DBIL Direct Bilirubin, TP Total Protein, ALB, Albumin, GLOB Globulin, AG Albumin-to-globulin ratio,ALT alanine aminotransferase, AST Aspartate transaminase, SHR Stress hyperglycemia.

| **Supplemental Table 3. Input variables for machine learning model development for secondary outcome** | |
| --- | --- |
| **Demographic characteristics** | Male  Age  Weight |
| **Vascular Risk factors** | History of heart failure |
|  | Hyperlipidemia |
|  | Systolic pressure |
|  | Smoking |
| **Stroke Clinical Characteristics** | Discharge NIHSS |
|  | TOAST classification |
| **Laboratory findings** | White blood cell count |
|  | Lymphocyte count |
|  | Neutrophil count |
|  | Blood platelet count |
|  | TG |
|  | TBIL |
|  | DBIL |
|  | ALB |
|  | GLOB |
|  | AG |
|  | AST |
|  | SHR |
|  | Hypersensitive C-reactive protein |

Abbreviations: TOAST Trial of ORG 10172 in Acute Stroke Treatment, NIHSS NIH Stroke Scale score, TG Triglyceride, TBIL Total bilirubin, DBIL Direct Bilirubin, ALB, Albumin, GLOB Globulin, AG Albumin-to-globulin ratio, AST Aspartate transaminase, SHR Stress hyperglycemia.

**Supplemental Table 4 Descriptive Analysis After MICE Imputation**

| **Characteristic** | **Original Data** | **Imputed Data** | **P Value** |
| --- | --- | --- | --- |
| White blood cell count | 7.43 (6.06, 9.15) | 7.67 (6.37, 8.86) | 0.3 |
| Unknown | 199 | 0 |  |
| Lymphocyte count | 1.69 (1.31, 2.12) | 1.78 (1.38, 2.18) | 0.014 |
| Unknown | 206 | 0 |  |
| Neutrophil count | 4.99 (3.85, 6.50) | 5.10 (4.02, 6.19) | 0.5 |
| Unknown | 206 | 0 |  |
| RBC | 4.53 (4.21, 4.84) | 4.55 (4.24, 4.84) | 0.5 |
| Unknown | 199 | 0 |  |
| Blood platelet count | 171 (13, 232) | 166 (18, 226) | >0.9 |
| Unknown | 206 | 0 |  |
| Hemoglobin | 140 (122, 157) | 142 (124, 162) | 0.068 |
| Unknown | 206 | 0 |  |
| CHOL | 4.88 (4.15, 5.67) | 4.89 (4.16, 5.67) | >0.9 |
| Unknown | 24 | 0 |  |
| HDL-C | 0.98 (0.86, 1.15) | 0.98 (0.86, 1.15) | >0.9 |
| Unknown | 24 | 0 |  |
| LDL-C | 3.25 (2.67, 3.74) | 3.25 (2.67, 3.73) | >0.9 |
| Unknown | 24 | 0 |  |
| TG | 1.55 (1.10, 2.22) | 1.56 (1.11, 2.24) | 0.8 |
| Unknown | 25 | 0 |  |
| TBIL | 15 (12, 20) | 15 (12, 20) | >0.9 |
| Unknown | 142 | 0 |  |
| DBIL | 2.60 (2.00, 3.60) | 2.60 (2.00, 3.50) | >0.9 |
| Unknown | 143 | 0 |  |
| TP | 66.5 (62.9, 70.5) | 66.5 (63.3, 69.9) | >0.9 |
| Unknown | 142 | 0 |  |
| ALB | 40.8 (38.7, 43.2) | 40.9 (39.1, 42.9) | 0.8 |
| Unknown | 142 | 0 |  |
| GLOB | 25.6 (22.7, 28.7) | 25.6 (23.0, 28.3) | >0.9 |
| Unknown | 144 | 0 |  |
| AG | 1.61 (1.40, 1.82) | 1.62 (1.43, 1.79) | 0.5 |
| Unknown | 149 | 0 |  |
| ALT | 19 (13, 26) | 19 (13, 26) | 0.5 |
| Unknown | 94 | 0 |  |
| AST | 19 (16, 24) | 19 (16, 24) | 0.7 |
| Unknown | 91 | 0 |  |
| Creatinine | 68 (57, 81) | 68 (57, 81) | 0.8 |
| Unknown | 65 | 0 |  |
| Blood urea nitrogen | 5.60 (4.58, 6.76) | 5.60 (4.61, 6.73) | 0.8 |
| Unknown | 65 | 0 |  |
| Hypersensitive C-reactive protein | 2.3 (1.0, 4.8) | 3.8 (2.3, 6.5) | <0.001 |
| Unknown | 727 | 0 |  |
| DL-Homocysteine | 15 (12, 20) | 15 (12, 20) | 0.3 |
| Unknown | 157 | 0 |  |
